# Modified Fasting Compared to True Fasting Improves Blood Glucose Levels and Subjective Experiences of Hunger, Food Cravings and Mental Fatigue, But Not Cognitive Function: Results of an Acute Randomised Cross-Over Trial

**DOI:** 10.3390/nu13010065

**Published:** 2020-12-28

**Authors:** Ian Zajac, Danielle Herreen, Hugh Hunkin, Genevieve James-Martin, Mathilde Doyen, Naomi Kakoschke, Emily Brindal

**Affiliations:** 1 Nutrition & Health, Health & Biosecurity, CSIRO, Adelaide, SA 5000, Australia; danielle.herreen@csiro.au (D.H.); hugh.hunkin@csiro.au (H.H.); Genevieve.James-Martin@csiro.au (G.J.-M.); mathilde.doyen@csiro.au (M.D.); naomi.kakoschke@csiro.au (N.K.); emily.brindal@csiro.au (E.B.); 2 School of Psychology, University of Adelaide, Adelaide, SA 5000, Australia; 3 Agrocampus Ouest, 65 Rue de Saint-Brieuc, 35000 Rennes, France

**Keywords:** fasting, weight loss, cognitive function, fatigue, satiety

## Abstract

Recent dietary trends have prompted growing support for a variety of fasting paradigms involving extreme restriction or nil-caloric intake on fasting days. Some studies indicate that fasting may negatively influence factors including cognitive function through inducing fatigue, which may prove problematic in the context of completing a range of cognitively demanding activities required by daily obligations such as work. This randomised within-subjects cross-over trial explored the effects of true fasting (i.e., nil-caloric intake) versus modified fasting, the latter of which involved two sub-conditions: (1) extended distribution (three small meals distributed across the day; 522 kcal total); and (2) bulking (two meals eaten early in the day; 512 kcal total) over a period of 7.5 h on a single day with a 7-day washout period between conditions. Participants were *n* = 17 females (Body Mass Index (BMI) Mean (M) = 25.80, Standard Deviation (SD) = 2.30) aged 21–49 years. Outcomes included cognitive function, subjective mental fatigue, satiety, food cravings and blood glucose. Results showed that there were no differences in cognitive test performance between conditions;however, both modified fasting sub-conditions had improved blood glucose levels, cravings, hunger and fullness compared to true fasting. Moreover, subjective mental fatigue was significantly reduced in the modified fasting conditions relative to true fasting. Overall, results indicated that the subjective experience of true fasting and modified fasting is different, but that cognition does not appear to be impaired.

## 1. Introduction

The number of adults with overweight or obesity has reached epidemic proportions [1]. Between 1975 and 2016, the prevalence of obesity increased significantly in every country, with a global average of 10.8% of men and 14.9% of women with a body mass index (BMI) ≥ 30 kg/m² [2]. Excess weight significantly increases the risk of developing a wide range of health conditions including hypertension, major cardiovascular disease, type II diabetes, psychological illnesses, some forms of cancer, and contributes to premature death [2]. In addition, health-related problems associated with excess weight impose substantial societal and economic burdens on individuals, families, and communities, resulting in one of the greatest public health challenges confronting many countries [3].

Losing weight by means of dietary restriction combined with energy expenditure from physical activity has been shown to combat the health risks associated with excess weight [4] and provides numerous benefits for emotional health and quality of life [5]. Continuous or daily energy restriction is the first-line therapy prescribed to individuals wishing to lose weight [6]. However, many individuals find the rigidity of daily calorie restriction too difficult to maintain [7]. As a result, alternate day fasting (ADF) regimes are increasingly promoted as a potentially superior alternative to daily caloric restriction. ADF diets typically involve a “fast day”, where energy intake is completely withheld, followed by a “feed day”, where food is consumed ad libitum [7,8]. This approach operates under the assumption that behavioural compliance increases as a result of a restrict-then-relax approach.

A complementary trend that has gained popularity over the last decade is the 5:2 intermittent fasting method. Unlike traditional ADF programs, approaches such as the 5:2 avoid complete fasting days, opting instead for days of extreme caloric restriction (up to 75% reduction). The 5:2 method involves five ad libitum eating days interspersed by two restricted calorie intake days of ~2100 kJ/day (500 kcal) for women and ~2500 kJ/day (600 kcal) for men. Consumer interest in this approach has resulted in commercially available intermittent fasting programs that utilise a combination of nutritionally balanced meal replacement shakes, soups, and bars to simplify calorie restriction days (for example, see Isagenix and Impromy Flexi). Although limited research has directly examined the 5:2 method to date, reductions in several biomarkers associated with the risk of developing type II diabetes and certain obesity-related cancers have been found in previous studies [9,10].

Despite the purported benefits of fasting approaches for weight loss, some previous studies have suggested that short-term fasting, at least in its true form (i.e., 0 kcal a day), may have acute negative effects including impaired cognitive function [11,12]. Specifically, a recent systematic review reported slower psychomotor speed, slower reaction times, reduced memory capacity, reduced working memory, and poorer inhibition and reasoning skills during a short-term fasted state [13]. Nevertheless, other studies have indicated that fasting has no impact [14,15] and overarching reviews have suggested that it may even enhance cognitive performance by instigating a “metabolic switch”; namely, a physiological shift from glucose to fatty acid utilisation, which improves brain health [16]. Given inconsistent previous research findings, it is necessary to gain a better understanding of the cognitive effects of fasting. Given the relatively acute phases of fasting in these dietary approaches, deficits that negatively impact daily activities—such as driving or employment, for example—through impaired cognitive function may be experienced as adverse effects which may have flow-on effects in relation to overall behavioural compliance with such patterns.

An important goal when implementing a weight loss strategy is to ensure high compliance and the observed, often negative, acute impact of true fasting may be problematic for factors including cognitive function. Importantly, few studies have considered any differential impacts of true fasting (0 kcal per day) as opposed to modified fasting (~500 kcal day). The purpose of the present study was to consider these differences in relation to measures of blood glucose, hunger, food cravings, mental fatigue, and cognitive performance. This study goes a step further to consider whether the timing of energy consumption (i.e., how restricted calories are distributed across the day) might also serve to minimise any negative effects, which has not previously been explored. The timing of energy consumption may be a factor that could abate any potentially negative effects of caloric restriction on cognitive function and hunger, particularly in the context of carbohydrates [17]. The primary reason relates to the impact of meal timing on metabolic measures such as blood glucose levels, and by disrupting the circadian rhythm and subsequently, hormone expression (e.g., cortisol). To test this idea, the present design involved comparing three alternate fasting conditions including (1) true fasting; (2) modified fasting—extended distribution (three small meals distributed across the day); and (3) modified fasting—bulking (two meals consumed early on in the day) in a sample of women who were healthy or overweight.

## 2. Materials and Methods

### 2.1. Design

**Figure 1:**
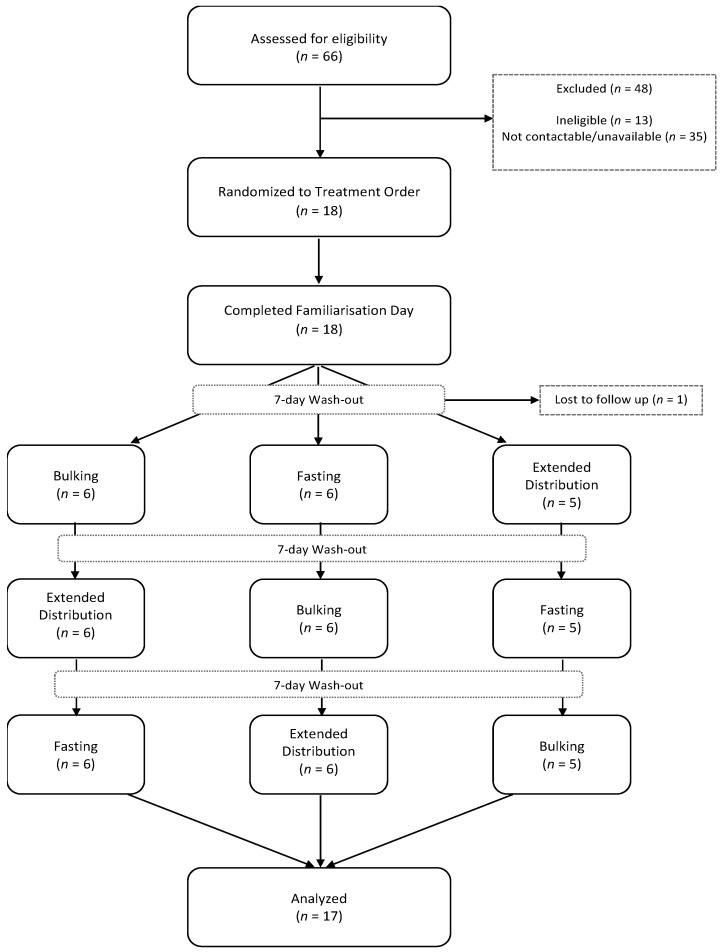
Participant flow through study.

This study used a randomised cross-over trial methodology, which incorporated three different fasting conditions, hourly-testing-times over a 7.5-h period (to examine differences across a single day) and a 7-day washout period between conditions (see Figure 1). All subjects gave their informed consent for inclusion before they participated in the study. The study was conducted in accordance with the Declaration of Helsinki, and the protocol was approved by the CSIRO Human Research Ethics Committee (ethical approval code: LR 16/2016). The trial was registered in a clinical trial registry (ACTRN12616001531404).

### 2.2. Participants

Participants completed an initial health screening questionnaire prior to enrolment in the study to determine eligibility. Inclusion criteria were: females aged 18 and over. Exclusion criteria were: BMI ≥ 30 (obese) or ≤18 (underweight), current smokers, diagnosis of diabetes, coeliac disease, uncontrolled hypertension, lactose intolerance, history of psychiatric disorders or neurological disease (e.g., dementia), and those with a nut or seed allergy. Eighteen (*n* = 18) female participants were recruited via advertisements on social media platforms. Participants were aged between 21 and 49 years (Mean (M) = 31.94, Standard Deviation (SD) = 9.10), with a mean BMI of 25.8 (SD = 2.30). All participants reported that they were in generally good health, had no allergy to dairy products, and were non-smokers.

### 2.3. Intervention

Given the focus of this study on fasting as a potential means to facilitate weight loss, a combination of commercially available liquid meal replacements (LMRs) as well as snack replacement bars (SRBs) was used. The fasting conditions included (1) true fasting (TF; 0 kcal); (2) modified fasting—(a) extended distribution (morning LMR plus two SRBs; 522 kcal); and (b) bulking (Two LMRs; 512 kcal). The composition of the LMRs and SRBs, including total nutritional intake for the conditions, is shown in Table 1.

(1) Extended Distribution Condition = 1 × Liquid Meal Replacement (LMR) + 2 × Snack Replacement Bar (SRB). Bulking condition = 2 × LMR. True Fasting Condition = nil intake (0 kcal).

### 2.4. Blood Glucose Measurement

Alcohol-soaked cleansing swabs were used for pre-sampling sterilisation of the skin. Blood samples were then obtained using Medlance Lite single-use capillary blood sampling lancets (HTL Strefa, Ozorków, Poland). Blood glucose levels were derived using the Accu-Chek Aviva Connect blood glucose sensor (Roche Diagnostics, Mannheim, Germany) and disposable Accu-Chek test strips. This system uses an enzymatic method of analysis which has been used in previous studies assessing blood glucose levels [18].

### 2.5. Satiety Questionnaire

Satiety and cravings were measured according to a validated subjective appetite sensations methodology [19], an approach adapted to measure the acute effects of interventions on these domains through the use of multiple repeated assessments, see e.g., [20]. Responses for satiety are elicited using four items assessing general hunger, satisfaction, fullness, and desire to eat. Cravings are measured with four items assessing desire for sweet, salty, savoury, and fatty foods. Each individual item is expressed as a visual analogue scale (VAS), 100 mm in length and anchored at each end with extremes (e.g., “Not at all full” and “Totally full”). Participants placed a mark on the VAS according to their subjective state. Scores for each item represent the distance (in millimetres) of the mark from the left anchor.

### 2.6. Cognitive Fatigue Battery

The Commonwealth Scientific and Industrial Research Organisation (CSIRO) Cognitive Fatigue Battery (C-CFB) is a purposefully comprised set of tasks designed to mentally strain participants over the course of several successive assessment runs and is operationally similar to cognitive fatigue batteries used in previous nutritional intervention studies [21]. The C-CFB is completed three consecutive times at each assessment point throughout the day, with no break in-between repeated runs. The battery broadly measured the domains of attention, response inhibition, psychomotor/response speed, and working memory. The tasks were programmed to run using the Inquisit platform.

#### 2.6.1. Mackworth Clock Task (MCT)

This task was used to measure sustained attention [22]. In this task, participants track the movement of a red probe (circle) around the circumference of a circle on the computer screen. The probe moves sequentially from point-to-point (24 points evenly dispersed around the perimeter). At random intervals, the probe skips a point (i.e., moves two spaces), and participants are required to respond when this happened by pressing the spacebar as quickly as possible. Ten skip-events are programmed to occur within the test’s three-min time limit. Accuracy is scored as percentage of skip-events correctly identified.

#### 2.6.2. Colour Multi-Source Interference Test (cMSIT)

The cMSIT [23] is a cognitive interference task measuring domains, including inhibition, psychomotor/response speed, and attention. In this task, stimuli consisting of three characters are presented in the centre of the computer screen. The characters are the numbers 1, 2, 3 or the letter “x”, depending on the trial. Participants must indicate as quickly and as accurately as possible the character that differs from the other two identical ones by pressing response keys labelled 1, 2 and 3, respectively. The MSIT consists of control trials, in which the target is always a number, the distractors are always the letter “x”, the target is larger than the distractors, and the target is always in its corresponding space (e.g., 1xx, x2x, xx3). In the interference trials, the target can be large or small in size and it does not have to match its position. Furthermore, the distractors in these trials are always other numbers (e.g., 331, 313, 121, 112 etc.). The difference in response times between interference and control trials for correctly answered trials is the MSIT effect. Attention to colour congruency is captured in a second outcome measure known as the attention effect, namely, the difference in reaction time between colour-congruent and colour-incongruent trials. Higher values on both measures represent greater inhibitory control during task performance.

#### 2.6.3. Serial Subtraction

This task taps into domains including attention and working memory and was adapted from previous studies [21]. Participants were briefly presented with a starting number between 800 and 999 before commencing, to count backwards for a duration of two minutes. Responses were recorded by mouse clicking a grid of numbers presented on the computer screen. Participants first subtracted by 3, then repeated the task subtracting by 7. The task was scored as the number of correct responses in each two-minute period.

### 2.7. Subjective Fatigue Visual Analogue Scales (VAS)

Participants indicated their current subjective state of fatigue using an original set of VAS adapted from existing tools such as the Bond–Lader VAS [24]. Items were single words associated with a VAS labelled “not at all” to “extremely”. Domains measured included Energy (five items), Sleepiness (four items), Alertness (four items), and Fatigue (four items). Participants indicated the intensity of their subjective state for each item by clicking on a 100 mm line on the computer screen.

### 2.8. Procedure

The study was advertised on the CSIRO Facebook page and a total of *n* = 66 volunteers completed the eligibility survey. Participants deemed eligible based on their responses were contacted via phone until *n* = 18 participants were enrolled, and then randomly allocated into three groups of six participants. Enrolled participants attended all testing days in the same group to which they were initially assigned. To balance carry-over effects between the fasting, bulking and extended-distribution conditions, participants were individually randomised to one of three counterbalanced treatment orders to balance carry-over effects (see Table 2 for assessment timing).

The first day of testing was a familiarisation day in which participants completed all study measurements (for all time-points) in order to remove practice effects on the cognitive battery and the subjective measures. Over the second, third and fourth testing days, participants completed the interventions as per their allocated order. Participants attended in a fasted state—confirmed via a blood glucose reading of ≤4.5 mmol/L—on a specific day of the week for four consecutive weeks to ensure a 7-day washout between visits. To assist with physical hunger pains, participants were provided with three small bags containing celery, capsicum and cucumber sticks totalling 525 g (100 kcal). Water was available ad libitum throughout the day, with no other beverages permitted. Prior to leaving each assessment day, participants consumed a small meal (i.e., sandwich and piece of fruit). Participants received $400 (Australian Dollars; AUD) for their participation upon completion of the study.

### 2.9. Power and Statistical Analysis

The number of participants required was predetermined using power analysis software (G*Power v3.1.9.6, Düsseldorf, Germany) [25]. Using the repeated measures within-between interaction effect module, it was determined that *n* = 45 participants would be sufficient to detect a small effect (f = 0.15, α = 0.05, β = 0.90), with high confidence assuming independent treatment groups. Importantly, this calculation does not consider the advantages of cross-over designs in relation to improved statistical power. Nevertheless, based on this model, we determined a minimum of *n* = 15 participants would be required to complete the study (i.e., 45 ÷ 3 = 15).

Statistical analyses were carried out with R v3.5.1 (Vienna, Austria) [26]. Exploratory factor analysis was used to extract common factors from the satiety questionnaire. Items assessing satiety converged on two factors representing domains of Hunger and Fullness which were strongly inversely correlated (r = −0.70). Craving items did not load consistently on either of these factors and were therefore analysed individually. Robust confirmatory factor analysis was used to extract parameters for the subjective fatigue scale. A satisfactory single-factor model (Confirmatory Factor Index (CFI) = 0.98, Tucker-Lewis Index (TLI) = 0.97, Root mean square error of approximation (RMSEA) = 0.068) representing global mental fatigue was achieved following the removal of two items (1× sleepiness, 1× alertness) based on model modification indices.

Within-participant correlations of study variables were computed to determine the extent to which pairs of variables covary within a participant, while excluding between-participant variability [27]. Change over time analyses were assessed in two ways. Firstly, response profile analysis models [28] were developed for outcome variables of interest. These models identified specific phases where the bulking and extended interventions departed significantly from the fasting intervention. To account for repeated measurements on the same participants, linear mixed effect models were used with a random intercept for each participant. Where likelihood ratio testing indicated a significant improvement to fit, a random linear time-of-day slope by participant was added, and/or a nested random intercept and time-of-day slope by intervention. For models of cognitive task measures, age and week of task were also controlled for. Model covariance matrices were unstructured except where there was substantial autocorrelation between residuals (Serial 3s and 7s), and in this case, an autoregressive (AR) (1) covariance structure was applied. Random effects and covariance matrices for each model are described in Table 3, Table 4 and Table 5. For the second change over time analysis, the area under the curve (AUC) for each intervention was calculated, which represents the cumulative effects of intervention differences across the day [28,29] relative to the fasting condition.

## 3. Results

One participant withdrew from the trial following the first familiarisation day, leaving a total of 17 participants who completed the trial and whose data were analysed. Within-participant correlations in change scores (i.e., changes over time within each variable) are presented in Table 3. There was a significant large negative correlation between hunger and fullness, as identified in the measurement model. Hunger had significant small-to-moderate positive correlations with all four cravings scores, while fullness showed the opposite trend. Hunger also had a weak negative relationship with blood glucose and a weak positive relationship with mental fatigue. Sweet, salty, savoury and fatty cravings all demonstrated small-to-moderate positive intercorrelations. Task measures from the cognitive fatigue battery were not correlated with blood glucose or subjective measures, but performance on the Serial 3s and Serial 7s tasks was moderately positively correlated. Lastly, performance on the MCT was positively correlated with Serial 3s and Serial 7s performance.

**Figure 2:**
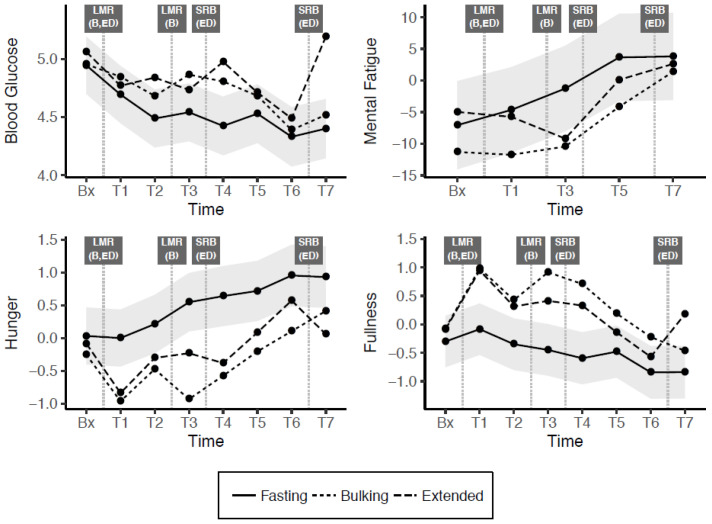
Change across the day in blood glucose, mental fatigue, hunger and fullness (shaded grey bands indicate 95% confidence interval (CI) for difference from fasting condition). Bx = baseline, T= Time(x), LMR = Liquid Meal Replacement, SRB = Snack Replacement Bar, B = Bulking, ED = Extended Distribution.

Model and AUC estimates for blood glucose, mental fatigue, hunger and fullness are presented in Table 4, while Figure 2 illustrates change in these outcome variables over time. In the fasting condition, blood glucose fell significantly throughout the day relative to baseline level. Compared to the fasting condition, blood glucose was significantly higher at several timepoints in the bulking (T3, T4) and extended (T2, T4, T7) conditions. Significant AUC differentials indicate that both modified fasting conditions resulted in cumulatively higher blood glucose levels throughout the day, relative to the true fasting group. In line with these findings, mental fatigue in the fasting group was significantly higher from baseline only at T5, while significant interactions showed a negative divergence in the bulking (T3, T5) and extended (T3) groups. AUC differentials indicated that across the day, this effect was significant, for the bulking group but not the extended group.

Hunger and fullness demonstrated opposing tendencies, as expected given the strong negative correlation between these two variables. In the true fasting condition, hunger increased significantly, and fullness decreased significantly relative to baseline by later timepoints. In comparison, there was significantly lower hunger and significantly higher fullness in both bulking and extended modified fasting conditions at most time points. These patterns were also reflected in the AUC differentials, all of which were significant and in the expected directions.

**Figure 3:**
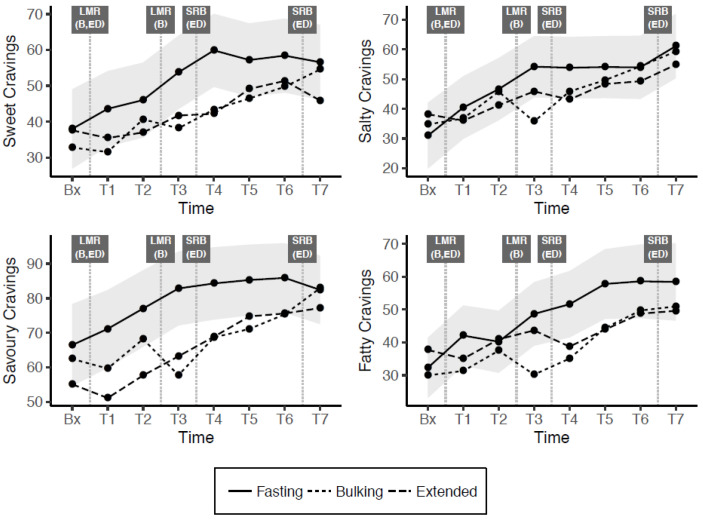
Change across the day in cravings (shaded grey bands indicate 95% confidence interval for difference from fasting condition). Bx = baseline, T= Time(x), LMR = Liquid Meal Replacement, SRB = Snack Replacement Bar, B = Bulking, ED = Extended Distribution.

Table 5 shows model and AUC estimates for sweet, salty, savoury and fatty cravings, which are depicted in Figure 3. In the fasting condition, these cravings tended to increase throughout the day, with significantly higher craving estimates for all interactions from T3 onwards. Cravings trended lower in the bulking and extended conditions, with the trajectory relatively similar between these conditions, and at least one timepoint across the day being significantly below the fasting level in all cases. AUC differentials indicated that the cumulative effect of these interactions was significant for sweet, savoury and fatty cravings (but not savoury cravings) in both the modified fasting (bulking and extended) conditions.

**Figure 4:**
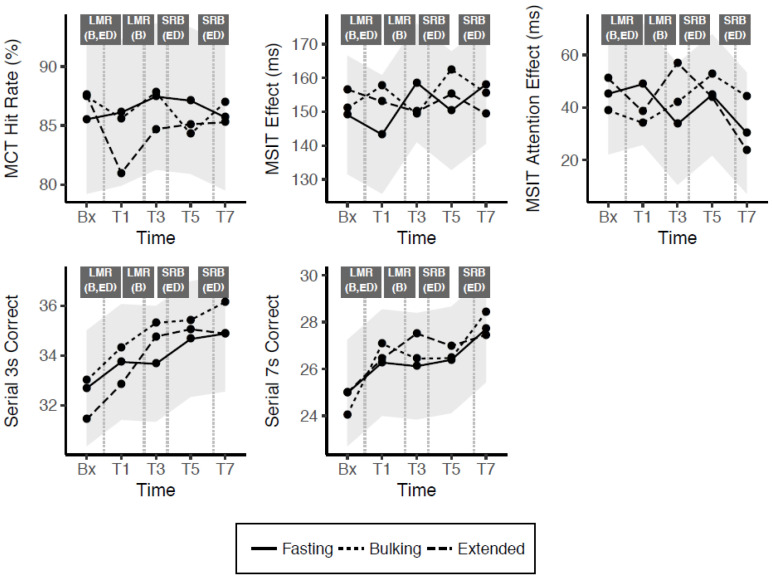
Change across the day in cognitive task outcome measures (shaded grey bands indicate 95% confidence interval for difference from fasting condition). Bx = baseline, T = Time(x), LMR = Liquid Meal Replacement, SRB = Snack Replacement Bar, B = Bulking, ED = Extended Distribution.

Model estimates and AUC parameters for tasks in the cognitive fatigue battery are presented in Table 6 and accompanied by Figure 4. A significant effect of trial week suggested that performance measured by the MCT and MSIT effect degraded over the three-week duration of the trial, whereas the number of successful serial counting trials increased. There were no main effects of time for any of the tasks, indicating that performance did not change significantly across the day in any condition. Additionally, the absence of significant treatment interactions suggests no difference between these cognitive performance outcomes across all conditions. In line with these results, AUC differentials were not significant for any combination of task and intervention.

Regarding the models described above and shown in Table 4, Table 5 and Table 6, additional hypothesis testing of model estimates was performed to consider any difference between modified fasting conditions. These results (presented as Supplementary Tables S1–S3) showed some limited differences between modified fasting conditions for outcomes including hunger, fullness, salty and fatty cravings. Furthermore, there were no differences between these in relation to cognitive performance.

## 4. Discussion

The present study appears to be the first to consider the acute effects of different fasting paradigms on measures of cognitive function, subjective mental fatigue, hunger, food cravings and blood glucose over a period of 7.5 h. In addition, the methodology also explored whether the timing of energy consumption (i.e., how restricted calories are distributed across the day) had any additional unique effects. Overall, both forms of modified fasting (500 Kcal) appeared to have generally improved most of the measured outcomes relative to true fasting (0 Kcal).

Within-participant correlations provide evidence of a small but significant relationship between lower blood glucose and higher subjective ratings of hunger, with the range of craving measures further implicated with rising hunger. Throughout the day, there were overall significant decreases in blood glucose, and fullness, and significant increases in fatigue and hunger for all forms of fasting. Modified fasting improved blood glucose, hunger and fullness, cravings and subjective mental fatigue over the study period relative to true fasting. There were no observable differences between conditions for any of the cognitive outcomes assessed.

In relation to the impact of fasting on measures of cognitive function, the literature to date appears mixed. Some studies have demonstrated acute negative effects, at least in relation to true fasting (0 kcal), resulting in impaired cognitive function [13], whilst others have indicated no impact of fasting on cognitive test performance [14,15]. The results of this study showed no acute effects of fasting throughout the day on cognitive function, with no change in performance for any cognitive measure across acute time points. Comparison of modified fasting to true fasting revealed no difference for any timepoints for any measure, with AUC models also showing no cumulative impact of modified fasting over the acute study periods. This is despite differences in blood glucose.

A possible relationship between dietary restriction and cognitive function has multiple plausible mechanisms. The first, more biologically focused theory suggests that fasting reduces blood glucose, which has downstream effects for cognition. For this to be supported, there would be an expected decrease in cognitive performance with lowering blood sugar levels and this would be observable in acute paradigms. This was not the case for our study. Despite overall higher blood glucose levels, there was no observable difference in mental performance. Other studies have manipulated blood glucose through varying glycaemic load to understand if it is the rise and fall in blood glucose rather than the specific value that could alter cognitive performance. However, this previous study also found minimal effects on hunger and mental performance despite observable differences in blood glucose (albeit in children) [30]. The tasks used in the current study may not have been demanding enough to see effects on blood glucose. Furthermore, despite good evidence for the facilitating effect of glucose ingestion on memory [31], it may be more difficult to observe deficits in cognitive performance resulting from low blood glucose levels.

An interesting finding in the present study is that changes in subjective fatigue were observed without associated changes in cognitive performance. This leads to another possible mechanism for how calorie restriction relates to cognitive function. An individual must have prolonged restriction in order to reduce their body weight. Conventional dieting typically requires 30–50% daily energy restriction over several months, which involves detailed monitoring and, for many people, learning counting schemes to self-monitor and comply. This monitoring results in high cognitive load and may explain why dieters have been observed as cognitively impaired in some domains [32]. In addition to this, psychological demands exist which could also create fatigue and mental impairment over the longer term. Continual mental effort to maintain a diet is thought to deplete the ego, which drains mental energy and willpower, making challenges more difficult to overcome. Strict restriction can also result in intrusive thoughts about food [33]. Multiple studies have shown that when hungry, individuals demonstrate a stronger attentional bias towards food-related cues [34,35]. Such biased processing may impair cognitive function in other areas. In the context of fasting, restriction is more extreme but also over a shorter period which allows dieters to take a break. It would be beneficial in future studies to explore the role of intrusive thoughts and ego depletion in fasting versus continual restriction diets. It would also be interesting to explore the downstream effects of mental fatigue across different types of restrictions.

Despite the strengths of this randomised, active cross-over trial, there are some limitations which should be considered in relation to the present findings. First, the sample size was small, although it was pre-determined using a power analysis calculator. Second, despite the intent that the cognitive battery herein would measure fatigue, the simplicity of the tasks within may have precluded this finding. The demands placed on cognitive functions throughout a typical day are likely to be greater than those imposed by the test battery, particularly when considering activities such as driving, and prolonged mental activity within the work environment, for example. Although not used in the present study, multi-tasking paradigms are designed to increase cognitive load and may prove fruitful in future investigations of the impact of fasting on cognitive function. In addition, the effects of the two modified fasting paradigms may be specific to the macronutrient composition of the meal replacement products used in the current study, given that the protein:carbohyrdate was higher than in a normal balanced diet. Future research should also aim to determine whether the highly acute effects observed here extend to longer-term effects. Finally, as we were interested in cognitive rather than metabolic outcomes, we excluded participants with obesity, which has been linked with impaired cognitive function [36]. Thus, the current results were obtained in healthy and overweight individuals rather than in those who are more likely to use meal replacements for weight loss, namely, individuals with obesity.

## 5. Conclusions

The present study has addressed ongoing uncertainty regarding the effects of fasting on cognitive function, as well as a range of additional outcomes relevant to the maintenance of fasting regimes for weight management. Based on the present findings, it is plausible to conclude that neither true fasting nor modified fasting negatively impact on cognitive function, at least for those outcomes measured herein. On the contrary, the subjective experience of modified fasting appears to be significantly different to true fasting in relation to hunger, food cravings, and perceived mental fatigue. Similarly, blood glucose was better maintained in the modified fasting conditions, and this appears to be one mechanistic driver of increasing hunger and food cravings. The benefits of these subjective improvements on modified fasting days should not be discounted. Indeed, they may prove significant regarding long term maintenance of fasting regimes for weight-management. Future studies should consider the extent to which the subjective experiences do predict longer term retention in such programs, and they should also explore the impact of fasting on more complex measures of cognitive function, such as driving and engagement in prolonged cognitive activities.

## Figures and Tables

**Table 1:** Nutritional information for study products.

Nutritional Information	Liquid Meal Replacement (LMR) ^1^	Snack Replacement Bar (SRB)
Weight (g)	276	35
Energy (kJ)	1071	555
Energy (cal)	256	133
Protein (g)	25.1	10.1
Fat (g)	4.1	5.4
Carb (g)	26.1	4.1
Fibre (g)	6.2	4.4
Weight (g)	276	35

^1^ Prepared in accordance with manufacturer specifications.

**Table 2:** Overview of assessment and nutritional intake timing.

Condition	Time												
	07:30	08:30	09:30	10:00	11:00	11:30	12:00	12:30	13:00	14:00	15:00	15:30	16:00
Bulking			LMR			LMR							
Extended			LMR					SRB				SRB	
Fasting	Nil Intake												
Assessments	Practice	Baseline		Time 1	Time 2		Time 3		Time 4	Time 5	Time 6		Time 7
	(SFS, CFB)	(SFS, CFB, BG, SQ)		(SFS, CFB, BG, SQ)	(BG, SQ)		(SFS, CFB, BG, SQ)		(BG, SQ)	(SFS, CFB, BG, SQ)	(BG, SQ)		(SFS, CFB, BG, SQ)

SFS = Subjective Fatigue Scales; CFB = Cognitive Fatigue Battery; BG = Blood Glucose; SQ = Satiety Questionnaire; LMR = Liquid Meal Replacement; SRB = Snack Replacement Bar.

**Table 3:** Within-participant correlations between change scores for all study variables.

Outcome Variables	1.	2.	3.	4.	5.	6.	7.	8.	9.	10.	11.	12.	13.
1. Blood glucose		−0.09	−0.14 *	0.10	−0.04	−0.01	0.01	0.00	0.00	0.05	0.00	0.06	0.07
2. Mental fatigue	−0.09		0.20 *	−0.17	0.13	0.05	0.15	0.10	0.02	0.09	0.05	−0.05	−0.03
3. Hunger	−0.14 **	0.20 **		−0.77 ***	0.21 ***	0.19 **	0.45 ***	0.26 ***	0.03	0.04	0.03	−0.13	−0.09
4. Fullness	0.10	−0.17 *	−0.77 ***		−0.12	−0.17 **	−0.36 ***	−0.25 ***	−0.07	0.00	0.04	0.11	0.12
5. Sweet craving	−0.04	0.13	0.21 ***	−0.12 *		0.31 ***	0.31 ***	0.21 ***	0.09	0.04	0.07	0.02	−0.05
6. Salty craving	−0.01	0.05	0.19 ***	−0.17 **	0.31 ***		0.37 ***	0.33 ***	0.08	−0.09	−0.06	0.09	0.08
7. Savoury craving	0.01	0.15 *	0.45 ***	−0.36 ***	0.31 ***	0.37 ***		0.24 ***	0.11	−0.04	−0.06	−0.02	0.09
8. Fatty craving	0.00	0.10	0.26 ***	−0.25 ***	0.21 ***	0.33 ***	0.24 ***		0.07	0.04	0.09	−0.04	−0.15
9. MCT	0.00	0.02	0.03	−0.07	0.09	0.08	0.11	0.07		0.02	0.07	0.27 ***	0.19 *
10. MSIT effect	0.05	0.09	0.04	0.00	0.04	−0.09	−0.04	0.04	0.02		−0.03	0.00	0.03
11. MSIT attention effect	0.00	0.05	0.03	0.04	0.07	−0.06	−0.06	0.09	0.07	−0.03		−0.03	−0.10
12. Serial 3s correct	0.06	−0.05	−0.13	0.11	0.02	0.09	−0.02	−0.04	0.27 ***	0.00	−0.03		0.46 ***
13. Serial 7s correct	0.07	−0.03	−0.09	0.12	−0.05	0.08	0.09	−0.15 *	0.19 **	0.03	−0.10	0.46 ***	

Note: *n* = 255 for mental fatigue and cognitive battery measures; for all other measures, *n* = 408; MCT = Mackworth Clock Task, MSIT = Colour Multi-Source Interference Test, Serial 3s and Serial 7s = Serial Subtraction Task (“subtract 3” and “subtract 7” conditions, respectively). Significance values indicated in upper triangle have been adjusted for multiple comparisons using the false discovery rate correction. * *p* < 0.05, ** *p* < 0.01, *** *p* < 0.001.

**Table 4:** Linear mixed effect model estimates and AUC differentials for blood glucose, mental fatigue, hunger and fullness.

Model Estimates	Blood Glucose (mmol/L)	Mental Fatigue	Hunger	Fullness
Fixed effects (B ± SE)				
(Intercept)	4.95 ± 0.12 ***	−7.07 ± 5.78	0.03 ± 0.20	−0.30 ± 0.22
Time: T1	−0.25 ± 0.11 *	2.41 ± 3.02	−0.03 ± 0.19	0.22 ± 0.20
Time: T2	−0.46 ± 0.11 ***		0.18 ± 0.20	−0.05 ± 0.20
Time: T3	−0.40 ± 0.12 **	5.79 ± 3.99	0.51 ± 0.21 *	−0.15 ± 0.21
Time: T4	−0.52 ± 0.12 ***		0.61 ± 0.22 **	−0.29 ± 0.22
Time: T5	−0.42 ± 0.12 **	10.72 ± 5.23 *	0.69 ± 0.23 **	−0.18 ± 0.23
Time: T6	−0.62 ± 0.12 ***		0.92 ± 0.24 ***	−0.54 ± 0.24 *
Time: T7	−0.55 ± 0.13 ***	10.88 ± 6.44	0.90 ± 0.25 ***	−0.54 ± 0.25 *
Bx × Bulking	0.01 ± 0.12	−4.24 ± 4.26	−0.28 ± 0.21	0.23 ± 0.24
T1 × Bulking	0.15 ± 0.12	−7.10 ± 3.87	−0.96 ± 0.21 ***	1.07 ± 0.23 ***
T2 × Bulking	0.20 ± 0.12		−0.69 ± 0.21 **	0.78 ± 0.23 **
T3 × Bulking	0.32 ± 0.12 *	−9.18 ± 3.56 *	−1.48 ± 0.22 ***	1.36 ± 0.23 ***
T4 × Bulking	0.38 ± 0.13 **		−1.21 ± 0.22 ***	1.30 ± 0.23 ***
T5 × Bulking	0.15 ± 0.13	−7.80 ± 3.46 *	−0.92 ± 0.23 ***	0.67 ± 0.23 **
T6 × Bulking	0.06 ± 0.13		−0.85 ± 0.23 ***	0.62 ± 0.23 **
T7 × Bulking	0.12 ± 0.13	−2.42 ± 3.53	−0.52 ± 0.24 *	0.38 ± 0.24
Bx × Extended	0.12 ± 0.12	2.09 ± 4.26	−0.12 ± 0.21	0.21 ± 0.24
T1 × Extended	0.08 ± 0.12	−1.10 ± 3.87	−0.84 ± 0.21 ***	1.03 ± 0.23 ***
T2 × Extended	0.35 ± 0.12 **		−0.52 ± 0.21 *	0.66 ± 0.23 **
T3 × Extended	0.19 ± 0.12	−7.92 ± 3.56 *	−0.78 ± 0.22 ***	0.86 ± 0.23 ***
T4 × Extended	0.55 ± 0.13 ***		−1.02 ± 0.22 ***	0.93 ± 0.23 ***
T5 × Extended	0.18 ± 0.13	−3.54 ± 3.46	−0.63 ± 0.23 **	0.34 ± 0.23
T6 × Extended	0.16 ± 0.13		−0.38 ± 0.23	0.27 ± 0.23
T7 × Extended	0.79 ± 0.13 ***	−1.19 ± 3.53	−0.87 ± 0.24 ***	1.02 ± 0.24 ***
Random effects (SD)				
Participant				
(Intercept)	0.35	20.50	0.56	0.57
Time (hrs)	0.03	2.84	0.07	0.07
Participant × Session				
(Intercept)	0.13	9.70	0.27	0.41
Time (h)	0.01	1.20	0.04	0.04
Residual	0.32	7.62	0.53	0.55
AUC differential (± SE)				
Bulking—Fasting	1.33 ± 0.47 **	−27.40 ± 10.30 **	−6.52 ± 0.91 ***	6.10 ± 0.97 ***
Extended—Fasting	1.97 ± 0.47 ***	−12.11 ± 10.30	−4.66 ± 0.91 ***	4.71 ± 0.97 ***

Note: Unstructured correlation matrices were used for all models. * *p* < 0.05, ** *p* < 0.01, *** *p* < 0.001. Bx = baseline; B = fixed effects estimate, SE = Standard Error; AUC = area under the curve.

**Table 5:** Linear mixed effect model estimates and AUC differentials for cravings.

Model Estimates	Sweet Craving	Salty Craving	Savoury Craving	Fatty Craving
Fixed effects (B ± SE)				
(Intercept)	37.99 ± 6.92 ***	30.98 ± 7.02 ***	66.44 ± 5.37 ***	32.30 ± 7.71 ***
Time: T1	5.60 ± 3.95	9.44 ± 3.81 *	4.69 ± 4.16	9.79 ± 3.98 *
Time: T2	8.05 ± 4.22	15.64 ± 4.10 ***	10.57 ± 4.38 *	7.92 ± 4.37
Time: T3	15.77 ± 4.46 ***	23.17 ± 4.36 ***	16.39 ± 4.54 ***	16.37 ± 4.79 **
Time: T4	21.90 ± 4.80 ***	22.84 ± 4.72 ***	17.85 ± 4.81 ***	19.31 ± 5.31 ***
Time: T5	19.22 ± 5.18 ***	23.05 ± 5.13 ***	18.84 ± 5.12 ***	25.47 ± 5.88 ***
Time: T6	20.43 ± 5.61 ***	22.96 ± 5.58 ***	19.45 ± 5.46 ***	26.28 ± 6.50 ***
Time: T7	18.60 ± 5.98 **	30.12 ± 5.97 ***	15.97 ± 5.76 **	26.11 ± 7.03 ***
Bx × Bulking	−5.20 ± 5.65	3.75 ± 5.66	−4.01 ± 6.06	−2.31 ± 4.69
T1 × Bulking	−12.04 ± 5.38 *	−3.65 ± 5.39	−11.49 ± 5.75 *	−10.75 ± 4.68 *
T2 × Bulking	−5.37 ± 5.34	−0.87 ± 5.35	−8.85 ± 5.66	−2.72 ± 4.83
T3 × Bulking	−15.60 ± 5.23 **	−18.43 ± 5.27 **	−25.11 ± 5.47 ***	−18.44 ± 4.94 ***
T4 × Bulking	−16.48 ± 5.20 **	−7.99 ± 5.28	−15.64 ± 5.35 **	−16.52 ± 5.15 **
T5 × Bulking	−10.73 ± 5.22 *	−4.39 ± 5.33	−14.18 ± 5.24 **	−13.41 ± 5.42 *
T6 × Bulking	−8.67 ± 5.27	0.33 ± 5.43	−10.55 ± 5.14 *	−8.88 ± 5.73
T7 × Bulking	−2.02 ± 5.34	−1.80 ± 5.54	0.53 ± 5.07	−7.47 ± 6.01
Bx × Extended	−0.40 ± 5.65	7.21 ± 5.66	−11.35 ± 6.06	5.42 ± 4.69
T1 × Extended	−8.15 ± 5.38	−4.42 ± 5.39	−20.02 ± 5.75 **	−7.05 ± 4.68
T2 × Extended	−9.03 ± 5.34	−5.41 ± 5.35	−19.38 ± 5.66 **	0.76 ± 4.83
T3 × Extended	−12.05 ± 5.23 *	−8.31 ± 5.27	−19.63 ± 5.47 ***	−5.02 ± 4.94
T4 × Extended	−17.68 ± 5.20 **	−10.72 ± 5.28 *	−15.44 ± 5.35 **	−12.87 ± 5.16 *
T5 × Extended	−8.07 ± 5.22	−5.69 ± 5.33	−10.54 ± 5.24 *	−13.87 ± 5.42 *
T6 × Extended	−7.04 ± 5.27	−4.68 ± 5.43	−10.29 ± 5.14 *	−9.85 ± 5.73
T7 × Extended	−10.68 ± 5.34 *	−6.27 ± 5.54	−5.24 ± 5.07	−8.80 ± 6.01
Random effects (SD)				
Participant				
(Intercept)	22.59	23.06	12.99	27.89
Time (h)	2.03	2.01	1.96	2.63
Participant × Session				
(Intercept)	11.95	12.37	12.86	8.26
Time (hrs)	1.22	1.37	0.78	1.59
Residual	10.66	10.22	11.37	10.38
AUC differential (± SE)				
Bulking—Fasting	−72.50 ± 26.18 **	−34.02 ± 27.53	−87.55 ± 26.61 **	−75.61 ± 24.99 **
Extended—Fasting	−67.54 ± 26.18 **	−38.76 ± 27.53	−103.60 ± 26.62 ***	−49.59 ± 24.98 *

Note: Unstructured correlation matrices were used for all models. * *p* < 0.05, ** *p* < 0.01, *** *p* < 0.001.

**Table 6:** Linear mixed effect model estimates and AUC differentials for cognitive fatigue battery tests.

Model Estimates	MCT Hit Rate (%)	MSIT Effect (ms)	MSIT Attention Effect (ms)	Serial 3s Correct Trials ^a^	Serial 7s Correct Trials ^a^
Fixed effects (B ± SE)					
(Intercept)	92.74 ± 8.69 ***	201.64 ± 21.33 ***	38.13 ± 17.89 *	39.65 ± 6.06 ***	29.93 ± 6.56 ***
Time: T1	0.58 ± 3.19	2.06 ± 8.96	−6.32 ± 11.78	1.06 ± 0.85	1.29 ± 0.86
Time: T3	1.93 ± 3.26	7.56 ± 8.96	5.85 ± 11.78	0.98 ± 1.04	1.14 ± 1.03
Time: T5	1.58 ± 3.38	−5.80 ± 8.96	3.69 ± 11.78	1.98 ± 1.12	1.41 ± 1.10
Time: T7	0.17 ± 3.52	8.65 ± 8.96	−11.06 ± 11.78	2.20 ± 1.16	2.73 ± 1.13
Age	0.02 ± 0.24	−0.66 ± 0.59	0.16 ± 0.42	−0.34 ± 0.18	−0.37 ± 0.19
Week	−2.61 ± 0.71 ***	−10.45 ± 2.01 ***	0.67 ± 2.64	1.31 ± 0.38 **	2.24 ± 0.37 ***
Bx × Bulking	1.94 ± 3.17	3.96 ± 8.96	−6.77 ± 11.78	0.31 ± 1.19	−0.93 ± 1.16
T1 × Bulking	−0.50 ± 3.17	7.48 ± 8.96	−5.18 ± 11.78	0.59 ± 1.19	0.80 ± 1.16
T3 × Bulking	0.36 ± 3.17	−7.35 ± 8.96	−9.04 ± 11.78	1.65 ± 1.19	0.33 ± 1.16
T5 × Bulking	−2.83 ± 3.17	6.75 ± 8.96	8.03 ± 11.78	0.76 ± 1.19	0.07 ± 1.16
T7 × Bulking	1.29 ± 3.17	−7.37 ± 8.96	10.70 ± 11.78	1.27 ± 1.19	0.72 ± 1.16
Bx × Extended	2.07 ± 3.22 ^b^	13.47 ± 8.96	7.43 ± 11.78	−1.25 ± 1.19	0.01 ± 1.16
T1 × Extended	−5.14 ± 3.17	4.08 ± 8.96	5.06 ± 11.78	−0.90 ± 1.19	0.16 ± 1.16
T3 × Extended	−2.76 ± 3.17	1.48 ± 8.96	−20.79 ± 11.78	1.10 ± 1.19	1.40 ± 1.16
T5 × Extended	−2.00 ± 3.17	12.27 ± 8.96	−4.63 ± 11.78	0.39 ± 1.19	0.59 ± 1.16
T7 × Extended	−0.42 ± 3.17	−8.34 ± 8.96	−10.43 ± 11.78	0.00 ± 1.19	−0.25 ± 1.16
Random effects (SD)					
Participant					
(Intercept)	16.60	20.28	12.48	6.31	6.91
Time (hrs)	0.81				
Residual	8.92	25.23	33.19	3.35	3.27
AUC differential (±SE)					
Bulking—Fasting	−1.35 ± 5.73	23.33 ± 19.35	−4.22 ± 21.30	3.79 ± 3.16	1.10 ± 3.02
Extended—Fasting	−9.08 ± 5.73	8.22 ± 19.35	−21.86 ± 21.30	−0.03 ± 3.16	2.03 ± 3.02

Note: ^a^ Covariance matrices were unstructured except for Serial 3s and 7s tasks, where an AR (1) structure was used (AR coefficient *ϕ* = 0.48 and 0.45, respectively). ^b^ Data from one participant was removed from this point as it formed an extreme outlier that did not meet assumptions for the MCT Hit Rate model. * *p* < 0.05, ** *p* < 0.01, *** *p* < 0.001.

## Data Availability

The data presented in this study are available on request from the corresponding author. The data are not publicly available due to privacy concerns.

## Supplementary Materials

The following are available online at https://www.mdpi.com/2072-6643/13/1/65/s1, Supplementary Table S1: Estimate of differences between bulking and extended distribution conditions for blood glucose, mental fatigue, hunger and fullness (supplements Table 4 in the main text); Supplementary Table S2: Estimate of differences between bulking and extended distribution conditions for cravings (supplements Table 5 in the main text); Supplementary Table S3: Estimate of differences between bulking and extended distribution conditions for cognitive fatigue battery tests (supplements Table 6 in the main text).
