# Supplementary material for: Modified Fasting Compared to True Fasting Improves Blood Glucose Levels and Subjective Experiences of Hunger, Food Cravings and Mental Fatigue, But Not Cognitive Function: Results of an Acute Randomised Cross-Over Trial
Source: Nutrients. 2020 Dec 28;13(1):65. doi: 10.3390/nu13010065 (PMC7824352; doi:10.3390/nu13010065)
Supplement: Supplementary file 1 [file nutrients-13-00065-s001.pdf]

Table S1. Estimate of differences between bulking and extended distribution conditions for blood glucose, mental fatigue, hunger, and fullness (supplements Table 4 in the main text).

Model Estimates	Blood Glucose (mmol/L)	Mental Fatigue	Hunger	Fullness
Fixed effects (B ± SE)				
Bx	0.11 ± 0.12	6.33 ± 4.14	0.17 ± 0.20	−0.02 ± 0.23
T1	−0.08 ± 0.12	6.00 ± 3.75	0.13 ± 0.21	−0.04 ± 0.23
T2	0.16 ± 0.12		0.17 ± 0.21	−0.11 ± 0.23
T3	−0.13 ± 0.12	1.26 ± 3.46	0.70 ± 0.21 **	−0.50 ± 0.22 *
T4	0.17 ± 0.12		0.19 ± 0.22	−0.38 ± 0.22
T5	0.03 ± 0.12	4.25 ± 3.35	0.29 ± 0.22	−0.33 ± 0.22
T6	0.10 ± 0.13		0.47 ± 0.23 *	−0.35 ± 0.23
T7	0.67 ± 0.13 ***	1.23 ± 3.43	−0.36 ± 0.23	0.64 ± 0.23 **

Note: Bx = Baseline, T= Time (x), B = fixed effects estimate, SE = Standard Error, mmol/L = millimoles per litre. Positive estimates indicate higher values in the extended distribution condition. * $p < 0.05$, ** $p < 0.01$, *** $p < 0.001$.

Table S2. Estimate of differences between bulking and extended distribution conditions for cravings (supplements Table 5 in the main text).

Model Estimates	Sweet Craving	Salty Craving	Savoury Craving	Fatty Craving
Fixed effects (B ± SE)				
Bx	4.81 ± 5.48	3.46 ± 5.49	−7.34 ± 5.87	7.73 ± 4.55
T1	3.89 ± 5.22	−0.77 ± 5.23	−8.53 ± 5.58	3.70 ± 4.54
T2	−3.66 ± 5.13	−4.54 ± 5.15	−10.53 ± 5.44	3.48 ± 4.63
T3	3.55 ± 5.07	10.12 ± 5.11 *	5.48 ± 5.31	13.42 ± 4.79 **
T4	−1.19 ± 5.05	−2.73 ± 5.12	0.19 ± 5.19	3.64 ± 5.00
T5	2.66 ± 5.06	−1.30 ± 5.17	3.63 ± 5.08	−0.46 ± 5.26
T6	1.63 ± 5.11	−5.01 ± 5.26	0.26 ± 4.98	−0.97 ± 5.55
T7	−8.66 ± 5.18	−4.46 ± 5.37	−5.77 ± 4.91	−1.33 ± 5.83

Note: Bx = Baseline, T= Time(x), *B* = fixed effects estimate, SE = Standard Error. Positive estimates indicate higher values in the extended distribution condition. * $p < 0.05$, ** $p < 0.01$, *** $p < 0.001$.

Table S3. Estimate of differences between bulking and extended distribution conditions for cognitive fatigue battery tests (supplements Table 6 in the main text).

Model Estimates	MCT Hit Rate (%)	MSIT Effect (ms)	MSIT Attention Effect (ms)	Serial 3s Correct Trials	Serial 7s Correct Trials
Fixed effects (B ± SE)					
Bx	0.13 ± 3.11	9.51 ± 8.66	14.20 ± 11.39	−1.57 ± 1.15	0.93 ± 1.12
T1	−4.64 ± 3.06	−3.41 ± 8.66	10.24 ± 11.39	−1.49 ± 1.15	−0.64 ± 1.12
T3	−3.13 ± 3.06	8.83 ± 8.66	−11.75 ± 11.39	−0.55 ± 1.15	1.07 ± 1.12
T5	0.83 ± 3.06	5.52 ± 8.66	−12.67 ± 11.39	−0.37 ± 1.15	0.52 ± 1.12
T7	−1.72 ± 3.06	−0.96 ± 8.66	−21.13 ± 11.39	−1.27 ± 1.15	−0.97 ± 1.12

Note: Bx = Baseline, T= Time(x), B = fixed effects estimate, SE = Standard Error.MCT = Mackworth Clock Task, MSIT = Colour Multi-Source Interference Test, Serial 3-s and Serial 7-s = Serial Subtraction Task ('subtract 3' and 'subtract 7' conditions, respectively). Positive estimates indicate higher values in the extended distribution condition. * p < 0.05, ** p < 0.01, *** p < 0.001.
